# Recent trends in water analysis triggering future monitoring of organic micropollutants

**DOI:** 10.1007/s00216-018-1015-9

**Published:** 2018-03-21

**Authors:** Torsten C. Schmidt

**Affiliations:** 1 Instrumental Analytical Chemistry, University of Duisburg-Essen, Universitätsstrasse 5, 45141 Essen, Germany; 2 Centre for Water and Environmental Research, University of Duisburg-Essen, Universitätsstrasse 5, 45141 Essen, Germany; 3 IWW Water Centre, Moritzstr. 26, 45476 Mülheim an der Ruhr, Germany

**Keywords:** Water analysis, Organic micropollutants, Effect-based analysis, High-resolution mass spectrometry, Multidimensional chromatography, Ion mobility

## Abstract

Water analysis has been an important area since the beginning of analytical chemistry. The focus though has shifted substantially: from minerals and the main constituents of water in the time of Carl Remigius Fresenius to a multitude of, in particular, organic compounds at concentrations down to the sub-nanogram per liter level nowadays. This was possible only because of numerous innovations in instrumentation in recent decades, drivers of which are briefly discussed. In addition to the high demands on sensitivity, high throughput by automation and short analysis times are major requirements. In this article, some recent developments in the chemical analysis of organic micropollutants (OMPs) are presented. These include the analysis of priority pollutants in whole water samples, extension of the analytical window, in particular to encompass highly polar compounds, the trend toward more than one separation dimension before mass spectrometric detection, and ways of coping with unknown analytes by suspect and nontarget screening approaches involving high-resolution mass spectrometry. Furthermore, beyond gathering reliable concentration data for many OMPs, the question of the relevance of such data for the aquatic system under scrutiny is becoming ever more important. To that end, effect-based analytics can be used and may become part of future routine monitoring, mostly with a focus on adverse effects of OMPs in specific test systems mimicking environmental impacts. Despite advances in the field of water analysis in recent years, there are still many challenges for further analytical research.

**Graphical abstract:**
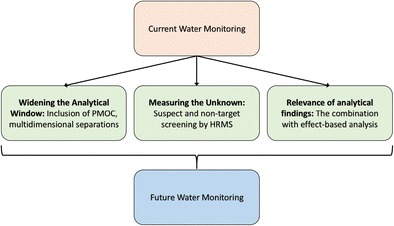
Recent trends in water analysis of organic micropollutants that open new opportunities in future water monitoring. HRMS high-resolution mass spectrometry, PMOC persistent mobile organic compounds

Recent trends in water analysis of organic micropollutants that open new opportunities in future water monitoring. HRMS high-resolution mass spectrometry, PMOC persistent mobile organic compounds

## Introduction

The primary task of water analysis is to provide information on the composition of aqueous samples of diverse origin. This has not really changed since the very beginning of analytical chemistry. To give just one example, Carl Remigius Fresenius, the godfather of analytical chemistry in Germany and founder of the journal that became *Analytical and Bioanalytical Chemistry*, specifically developed analytical methods for the investigation of mineral water from wells in the nineteenth century [1]. Beyond this well-established area of water analysis, in the past two centuries the main research interests have shifted dramatically from analysis of major ions. The focus is on reliable, fast, and extremely sensitive detection and quantitation of (micro)organisms, particles, and organic micropollutants (OMPs). In that sense, water analysis nowadays typically means measuring the unwanted to ensure good water quality. The further discussion will be limited to the latter class of compounds, which are often also referred to as “trace organic compounds” or “trace organic contaminants.” Since most of them are not yet regulated, they are often also classified as emerging contaminants or chemicals of emerging concern. An excellent overview of relevant compound classes in water analysis is given by Susan Richardson in her biannual reviews in *Analytical Chemistry* [2]. In the following, trends in water analysis of OMPs will be presented with a focus on widening the analytical window, the advent of high-resolution mass spectrometry (HRMS), and recent developments in effect-based analysis. Restriction of the scope of this article to OMPs should not be misinterpreted as a ranking of the importance for future developments in water analysis. In fact, from the viewpoint of human health and challenges, methods to better, faster, and more sensitively capture the hygienic status of water are still most relevant, in particular in low-income countries with less developed water supply management systems.

## Drivers in water analysis

In contrast to some other areas of measurement science, there are several drivers for further developments of methods for water analysis. The first is the same as in any area of science; that is, curiosity of the scientist, who in our case wants to find out if new instruments or new methods can be developed that improve existing approaches for monitoring water quality. The second is more specific for environmental analysis, and mostly concerns the sensitivity of analytical methods. On the basis of the precautionary principle or extrapolated risk estimates, a threshold limit for a certain pollutant or class of compounds is set that cannot or can hardly be achieved at the time of regulation enforcement. This triggers many developmental efforts because without means for control, such standards are useless. The best known example for this driver is the maximum allowable concentration of 0.1 μg/L set for pesticides and their metabolites in drinking water in the European Union (EU) in 1980. At that time, this value was considered to mean practically zero and was applied to all compounds of that class regardless of their toxicological evaluation. This precautionary value inspired a lot of work on pesticide residue measurements that nowadays allow the analysis of hundreds of compounds in a single multiresidue method with sensitivities in the low nanogram per liter range, thus surpassing the former “zero limit” by far [3]. As an example, Moschet et al. [4] reported a comprehensive assessment of Swiss surface water samples by the combined target analysis and suspect screening of more than 380 pesticides and their transformation products. Nowadays researchers are confronted with similar situations, for example, with regard to the extremely low environmental quality standard of 0.65 ng/L (annual average concentration, inland waters) for perfluorosulfonic acid and its derivatives [5] or the maximum accepted method detection limits of 0.035 and 0.4 ng/L indicated in the European watch list [6] for three estrogens that currently trigger many further analytical developments. A third driver is based on capturing the impacts of water composition on the system under scrutiny. We could also call it a “problem-based driver” for analytical developments. Again, this is not really new since methods demanded by ever-increasing quality requirements for water quality in manufacturing processes and the energy sector have been developed in recent decades that are able to achieve extreme sensitivities for certain analytes involved, for example, in corrosion processes. With regard to aquatic environments, we mostly refer to adverse impacts on organisms and ecosystems. In a few cases it has been shown that extremely low concentrations of specific compounds such as endocrine-disrupting compounds can have such impacts. In an already classic field experiment, Kidd et al. [7] added low amounts of the synthetic estrogen 17α-ethinylestradiol to one lake in an experimental lake system in Ontario, Canada. For 3 years, the concentration was kept at 5–6 ng/L in the lake. To test for effects they measured the biomarker vitellogenin in fathead minnow (*Pimephales promelas*) and found extremely high production in both female and male fish compared with production in a reference lake in the same system. Disturbance of reproduction over the 7-year observation period led to a collapse of the whole fish population in the lake with 17α-ethinylestradiol dosing [7].

## Are we always coping with the known?

The bottom line of water analysis in a European context is the EU Water Framework Directive (WFD) and derived regulations. As mentioned before for the recently added perfluorinated compounds and estrogens, there are priority substances or substance classes listed in Annex X and the watch list for which reliable methods at the required sensitivity applicable in routine monitoring are still not available. A further difficulty with the regulation can be the requirement of analysis of the whole, unfiltered water sample, including suspended particulate matter. Several years ago Lepom et al. [8] and Ademollo et al. [9] discussed analytical needs resulting from implementation of the WFD [8, 9], but progress since has in part been incremental, and there are still clear gaps. Only recently did methods become available that allow one-step analysis of some of the initial priority pollutants, including a number of legacy organochlorine pesticides and polybrominated diphenyl ethers, regardless of the content of suspended particulate matter and with the required sensitivity by the use of disk-based solid-phase extraction (SPE) and large-volume injection gas chromatography (GC)–mass spectrometry (MS) [10, 11]. In general, disk-based SPE is expected to become more relevant in the future, partly because of European standards explicitly requiring its use in WFD monitoring of specific substance classes. In the future any further amendment of the existing regulation requiring analysis of new compounds or at lower concentrations should be accompanied by mandated projects to establish analytical standard methods applicable in routine analysis.

## Widening the analytical window

**Fig. 1:**
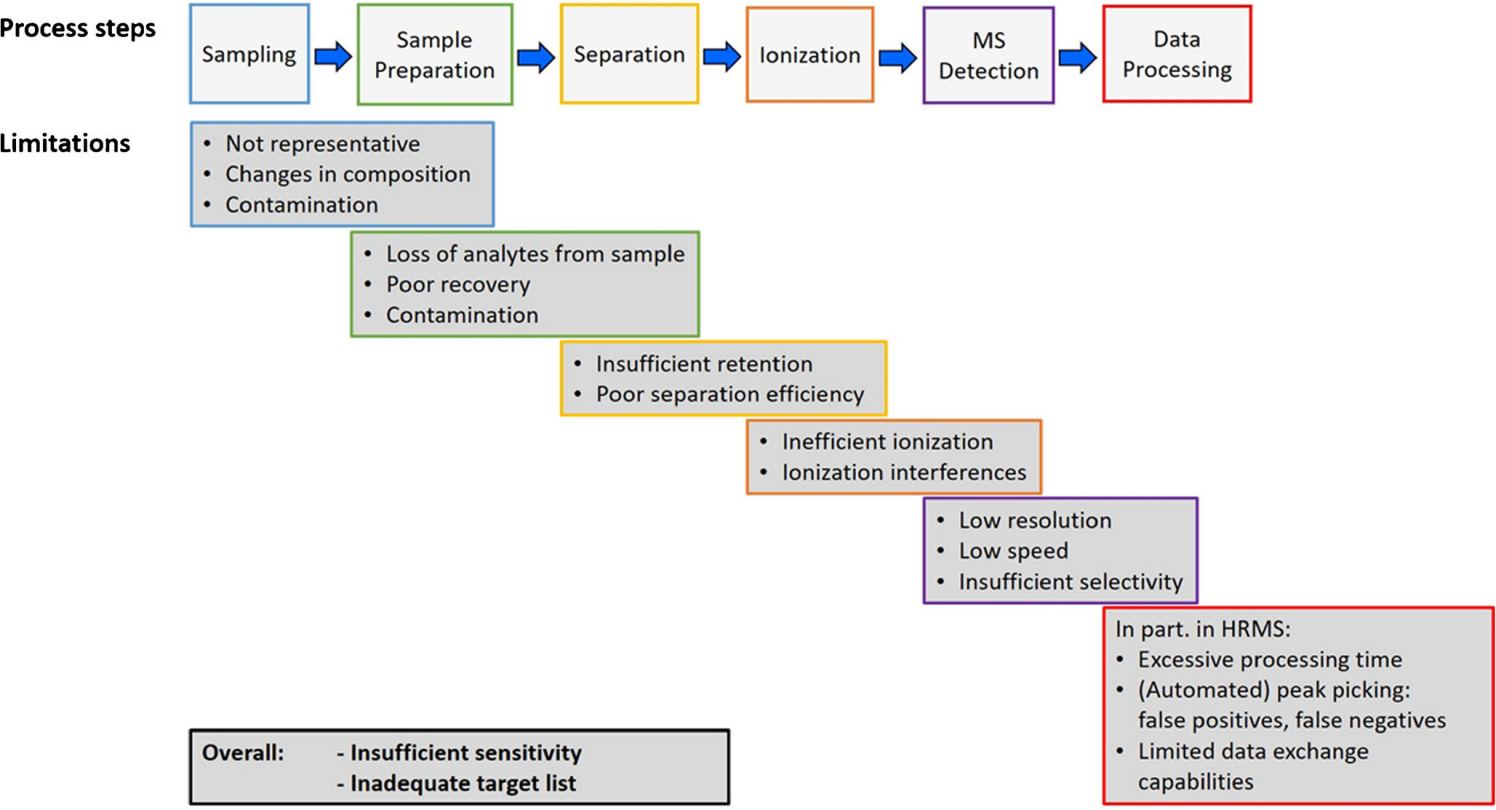
Potential reasons for the inherently incomplete analytical window in a generic analytical workflow using mass spectrometric (MS) detection. HRMS high-resolution mass spectrometry

As in all measurements one needs to accept that we will never be able to measure everything in a sample. The analytical window is inherently restricted for numerous reasons that are (incompletely) summarized for a generic analytical process based on chromatography coupled with MS detection in Fig. 1. In every single step there might be analytes that are excluded totally or partially because of the method used. Every practitioner in the field can surely add further points to that overview. Information loss in the huge amount of data processing and analysis shown in Fig. 1 may become the most critical issue in HRMS use (see later). In addition to the step-specific exclusions, the overall sensitivity of the final method may not be sufficient to detect compounds at the trace to ultratrace levels nowadays required for OMPs. In the case of target analysis, one may also have looked for the “wrong” targets (i.e., compounds that are not relevant in the system investigated), but disregarded inclusion of other, relevant compounds in the target lists. As long as a method is not validated for analysis of specific target analytes, an exclusion will remain mostly unnoticed.

**Fig. 2:**
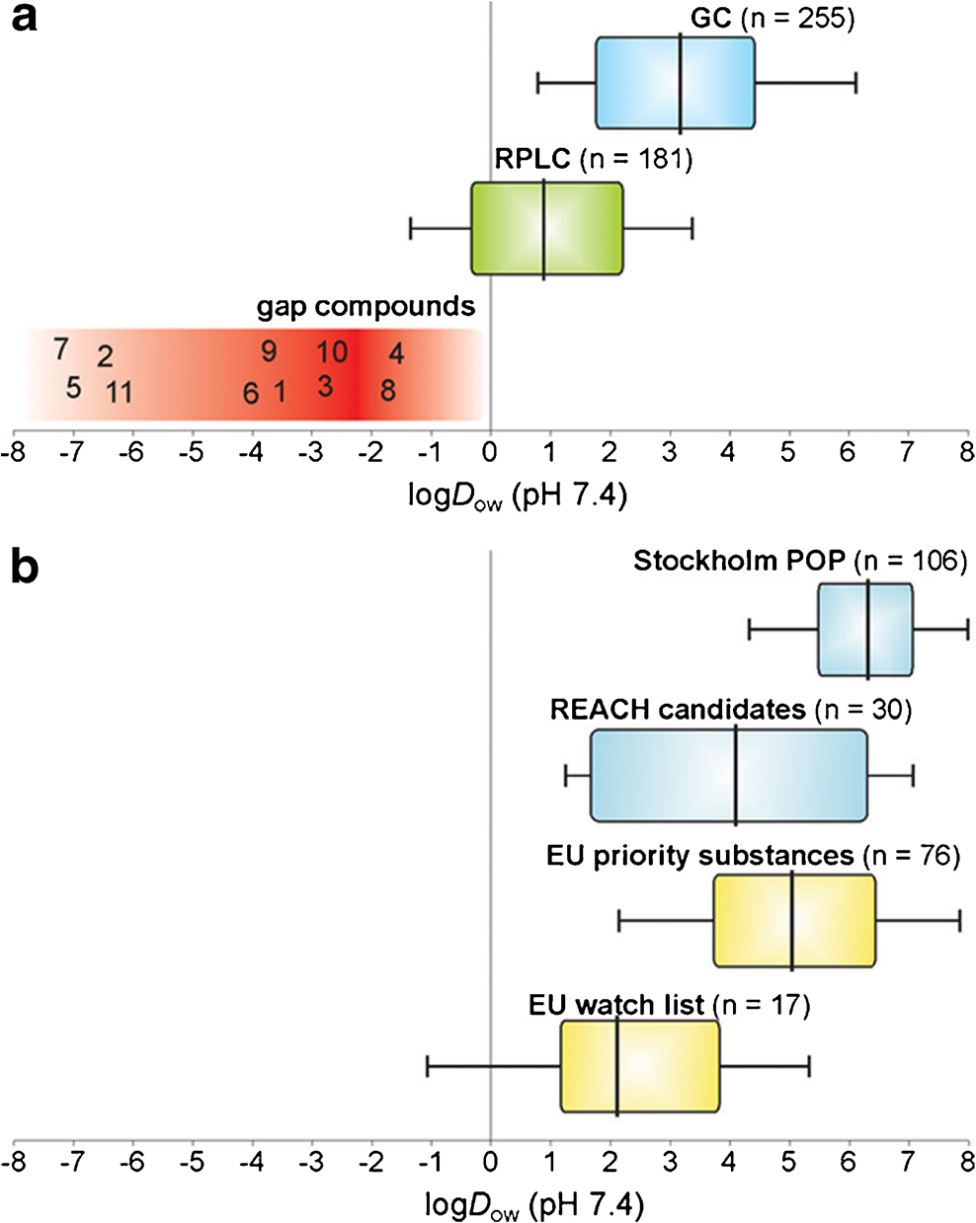
Classification of organic micropollutants with regard to their speciation-adjusted partitioning constants between octanol and water (*D*_ow_). Listed “gap compounds” represent persistent mobile organic compounds and comprise the following substances: aminomethylphosphonic acid (1), paraquat (2), cyanuric acid (3), *N*,*N*-dimethylsulfamide (4), diquat (5), 5-fluorouracil (6), glyphosate (7), melamine (8), metformin (9), trifluoroacetic acid (10), and EDTA (11). EU European Union, GC gas chromatography, POP persistent organic pollutant, REACH Registration, Evaluation, Authorisation and Restriction of Chemicals, RPLC reversed-phase liquid chromatography. (Reprinted with permission from [12]. Copyright 2016 American Chemical Society)

In the next two sections the focus is on widening the analytical window by further developments in the separation step, and subsequently the extended scope of HRMS detection will be discussed. In recent years, efforts in water analysis have been made with regard to a more comprehensive and reliable measurement of polar organic compounds. If these are in addition recalcitrant to degradation in the environment, they are the OMPs most likely to pass a multibarrier system and eventually reach raw water and even drinking water. The term “persistent mobile organic compounds” (PMOCs) was recently introduced to classify such compounds (Fig. 2). The state of the art and challenges in analysis of such compounds were summarized recently by Reemtsma et al. [12]. Besides the use of PMOCS as industrial chemicals, pharmaceuticals, or pesticides, many transformation reactions in technical and environmental systems also lead to more polar transformation products that add to the large diversity of PMOCs. Although one can argue about the range covered by reversed-phase high-performance liquid chromatography (RPLC) as indicated in Fig. 2, and there are means to enhance this range toward retention of more polar compounds, clearly there is a gap that cannot adequately be analyzed with established approaches. Therefore, other separation mechanisms are used, either solely or in combination with RPLC. These include ion chromatography, hydrophilic interaction liquid chromatography (HILIC), use of mixed-mode columns [13], supercritical fluid chromatography (SFC), and capillary electrophoresis, typically in combination with MS detection. Increasingly popular in that regard is HILIC as recently reviewed by Salas et al. [14], although at first sight it is not well suited for water analysis. This is because direct injection of large volumes of water as routinely done in RPLC of aqueous samples is impossible because of the high elution strength of water in HILIC. To avoid dilution of the sample with an organic solvent, most environmental applications so far have used SPE for sample preparation [5], of course with the risk of losing analytes in that step. An alternative that allows the analysis of polar and nonpolar compounds in one chromatographic run is the serial coupling of RPLC and HILIC as introduced by the group of Letzel [15]. One potential disadvantage is the need for a makeup flow of organic solvent after the first column, which may affect sensitivity, but successful application in suspect screening of OMPs in wastewater treatment plant effluents was shown. Bieber et al. [15] compared RPLC–HILIC with SFC using a single zwitterionic HILIC phase, and found complementary results with this approach [15]. The retention mechanisms in SFC are still not as well understood as in RPLC, but with increasing availability of robust instrumentation, SFC will gain popularity. Capillary electrophoresis would be perfectly suited for analysis of ionizable compounds. Limitations in the achievable sensitivities due to the small injection volumes applicable still prevent wider use, but capillary electrophoresis may provide a further alternative for the analysis of contaminated samples. Furthermore, in combination with in-line SPE for enrichment, method detection limits in the nanogram per liter range have already been reported [16], demonstrating its principal suitability for environmental samples. In the future the definition of a set of exemplary PMOCs for further method development would help enormously in comparison and evaluation of different approaches, even though it seems unlikely that a single method will be found to be superior in all cases.

## How many dimensions are enough in separation?

**Fig. 3:**
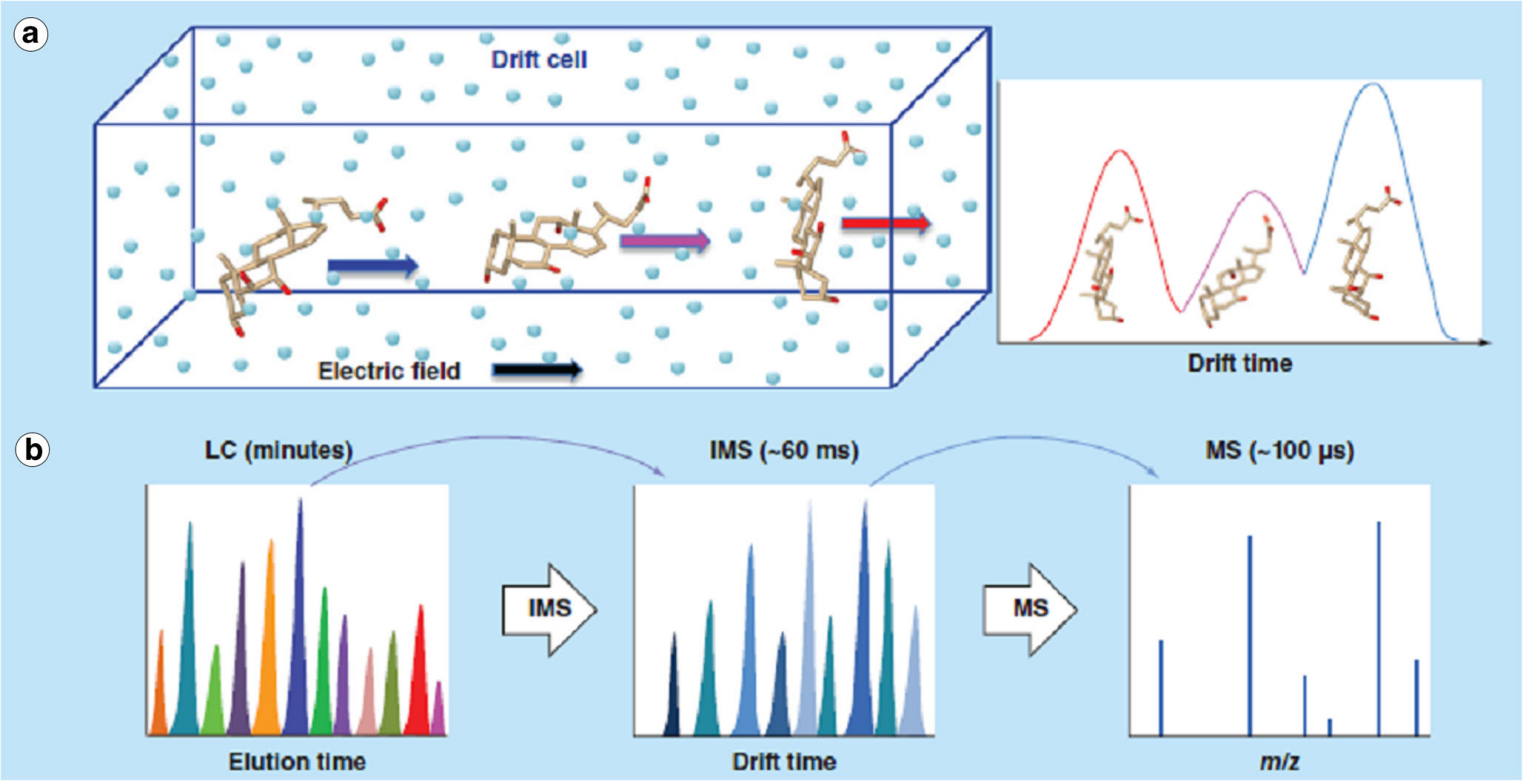
**a** Ion mobility separation based on the size and shape of molecules leading to different drift times. **b** Time domains of separation in liquid chromatography (LC), ion mobility spectrometry (IMS), and mass spectrometry (MS). (Reprinted with permission from [20] as agreed by Future Science Ltd.)

Water samples can contain complex mixtures of many OMPs at low concentrations. The peak capacity of a one-dimensional system may then not be sufficient to allow complete separation. Because of the selectivity of MS detection, this may be acceptable in many cases, and in nontarget approaches it is often impossible to locate any individual peak in the total ion current chromatogram. However, the poorer the separation, the more likely matrix effects or coelution of isobaric compounds occurs, which may adversely affect the analytical results, eventually leading to false positives or false negatives. Therefore, two-dimensional separations with the aspiration of orthogonality of separation mechanisms have been investigated also in the area of water analysis [17, 18]. Leonhardt et al. [19] compared one-dimensional and two-dimensional separations for the analysis of wastewater samples, and demonstrated that after two-dimensional separation a higher number of target compounds in a complex wastewater sample was always found than after one-dimensional separation [19]. One can even further increase the number of separation dimensions by implementing an additional ion mobility (IM) separation step before the MS detection. IM separation fits perfectly well in the time domain between separation by liquid chromatography (LC) and MS detection as visualized in Fig. 3, and may allow separation of isobaric compounds if their shape is sufficiently different to cause differences in drift time. This is already well established in bioanalysis but only recently has it been used in water analysis as well. Stephan et al. [21] demonstrated the potential for the investigation of wastewater with a four-dimensional system comprising two LC separation dimensions, IM separation, and MS detection [21]. Both multidimensional and IM separations will surely see further developments and applications soon, in particular in combination with HRMS detection in suspect and nontarget screening (see later).

## Measuring the unknown

**Fig. 4:**
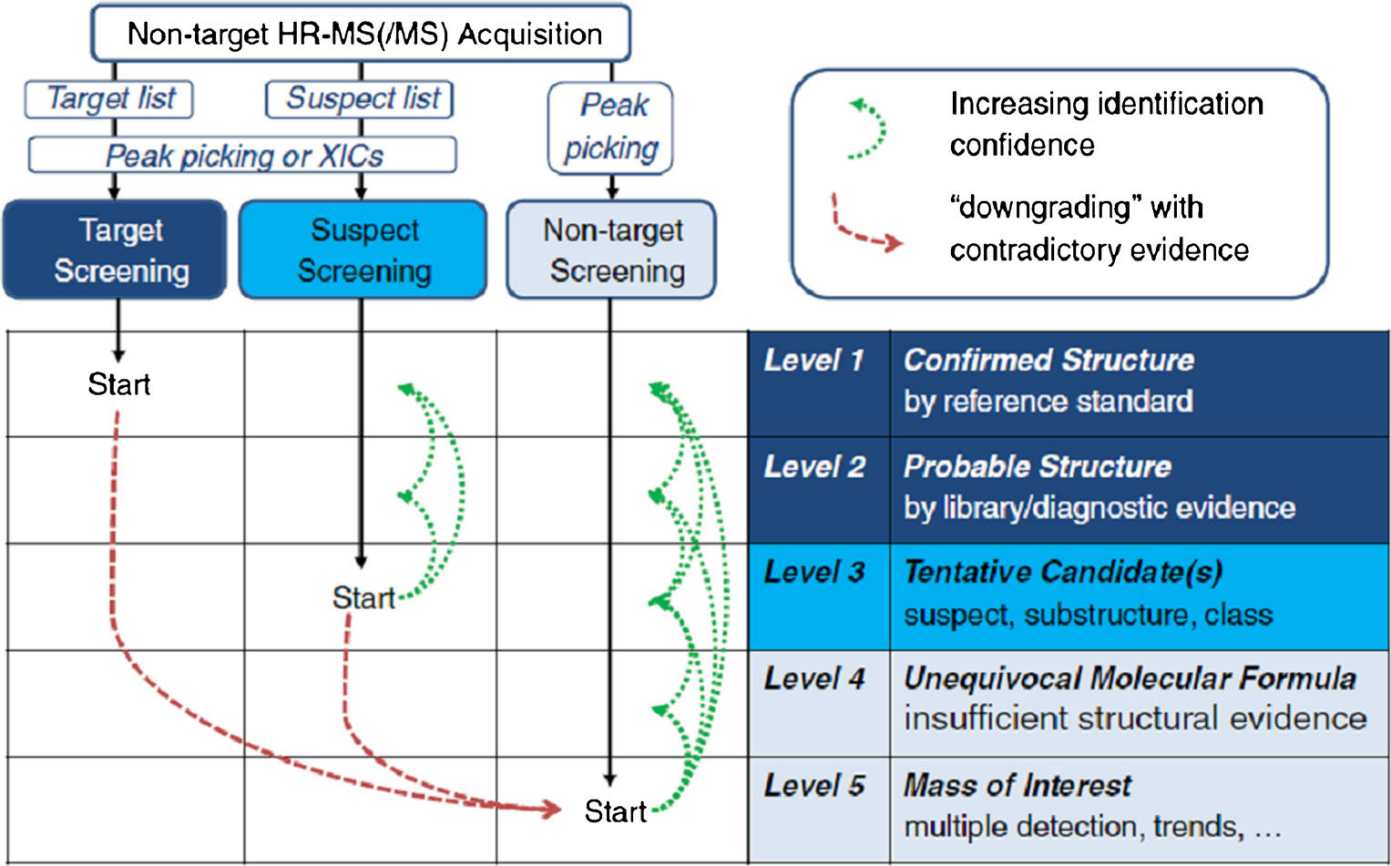
Identification approach involving high-resolution (HR) mass spectrometry (MS) combined with confidence levels of structural elucidation. *XIC* extracted ion chromatogram. (Reprinted from [26])

GC or LC separations in combination with MS and MS/MS detection, mostly based on low-resolution quadrupole analyzers, are well established for selective and sensitive detection of known target analytes. These include regulated compounds, for example, derived from implementation of the EU WFD or the US contaminant candidate list, but also an even larger number of unregulated or emerging compounds. A prerequisite is the availability of authentic reference standards and a meaningful a priori selection of target analytes for the samples investigated. Considering that already more than 1000 OMPs have been reported to occur in aquatic systems, a target analysis of all these compounds seems impossible in regular monitoring despite multiresidue protocols comprising hundreds of target or suspected compounds [3, 4, 22, 23]. Furthermore, for many organic compounds, including most metabolites and transformation products, there are currently no analytical reference standards available, rendering a target analysis impossible. At the same time, some regulated compounds are hardly detected anymore, for example, because of the phasing out of production, but still have to be monitored. This does not imply that there are fewer chemicals in our environment; we may just not measure the relevant ones. Although the EU acted on this problem by the implementation of a watch list for compounds of potential concern to be further investigated by the member states, the number of selected compounds will always be small and the selection process will always be time-consuming. Thus, it cannot fully reflect OMPs of recent concern. To overcome these limitations, HRMS detection based on time-of-flight or Orbitrap analyzers has become more popular in water analysis to allow suspect and nontarget screening without the need for reference standards. Although approaches using HRMS detection were discussed within the first decade of this century, the systematic classification of the two approaches in water analysis was introduced in the landmark article by Krauss et al. [24]. Since then, a confidence level scheme for structural elucidation of unknowns by HRMS has been suggested by Schymanski et al. [25] and has already been widely adopted. The general scheme with the five confidence levels and the relation to analytical workflows using HRMS is depicted in Fig. 4. A more detailed discussion of workflows in HRMS can be found in recent reviews [6, 27]. Furthermore, the results of a first interlaboratory collaborative trial have been published [26], which demonstrates that HRMS-based methods are out of their infancy already. Nevertheless, among other research needs, further harmonization of data processing has been emphasized. Data exchange among different software platforms, MS instrument suppliers, and open-source MS databases is still a major problem to be overcome in the future, and the latter have to be further developed and maintained. Finally, processing and management of huge amounts of data is a general challenge. All of these points apply not only to water analysis of course, and this area will surely benefit from advances in other areas, such as metabolomics, that use similar workflows. However, many examples demonstrate already the new opportunities created by HRMS use, including time and spatial trend analysis, even retrospectively, of features, process evaluation, and similarity analyses. For example, use of HRMS-based analytical methods may eventually lead to the detection and identification of so far unknown pollutants of relevance, as demonstrated for the river Rhine, where industrial emissions into the river or its tributaries were shown for the pharmaceutical tizanidine [28] and quaternary triphenylphosphonium compounds [29]. Further prospects of nontarget screening were discussed recently by Hollender et al. [30]. Because of the dominance of LC-based methods in today’s water analysis, all of the cited references focused on LC–HRMS. However, there are also a few examples that clearly show similar capabilities in combining GC and HRMS [31], and we will most likely see more interesting results from that hyphenation in the future.

## Is what we measure relevant after all?

Monitoring of OMPs in aqueous systems has little value as such, it serves as a basis for evaluation of aquatic systems or processes. Therefore, the question of what we can finally do with all the information from the widened analytical window obtained by extended separation methods and HRMS use becomes ever more important. For example, is advanced wastewater treatment for reduction of emissions by wastewater treatment plants meaningful or even necessary to improve the status of our surface waters? Feature numbers or target concentrations will not suffice to provide an answer to that question, or only in the rather few cases where human toxicity and ecotoxicity had been previously evaluated. The current debate on glyphosate genotoxicity highlights how difficult and sometimes controversial such an evaluation may be [32, 33]. Furthermore, mixture toxicities are not taken into account in such evaluations, so synergistic or antagonistic effects of compounds will not be captured. To fill this gap, effect-based analysis is a necessary complementary tool to chemical analysis and should be further implemented in future revisions of the EU WFD [34]. Wernersson et al. [35] summarized the current status of such an implementation based on an EU technical report. Effect-based analysis comprises both the directed measurements of specific end points, often in cell-based in vitro test systems, and the lumped effect evaluation on aquatic organisms with in vivo studies. The latter can then be combined with in situ observations in aquatic systems on the population level as mentioned earlier in the study of Kidd et al. [7]. The starting point in effect-directed analysis is in most cases the use of a cell-based bioassay test battery that encompasses relevant end points for aquatic systems [36]. Sometimes in addition or instead a standardized in vivo test, mostly with just a few aquatic species, such as *Daphnia magna*, *Danio rerio* (fish eggs or embryos), or different species of microalgae, is used. When using in vivo test systems, one should include taxa of different trophic levels [37]. In the tests either the investigated water itself or extracts from an enrichment step (e.g., by SPE) are used. In the latter case, negative control samples are very important to avoid false positive results caused by procedural blanks. Of course, the sample pretreatment chosen may also affect the final results since (unknown) toxic compounds might be lost or at least not recovered completely in the process [36]. Careful validation with a set of compounds of different properties should therefore be mandatory but is sometimes not done or at least not reported appropriately. If effects in one or more of the bioassays are found, this can be used for an effect-based evaluation of water samples and/or further prioritization of chemical analysis. As a result of a comprehensive study including 20 laboratories and more than 100 in vitro bioassays, Escher et al. [38] showed that (1) bioassays can be used to compare different water samples ranging from wastewater to reverse osmosis filtrate with regard to adverse effects and (2) recommendations for end points to be included in future test batteries for routine monitoring can be given. These should at least cover induction of the xenobiotic metabolism, endocrine disruption, and adaptive stress response. For measurement of end points indicating rather nonspecific effects, only a small fraction (typically less than 1%) of the effect can currently be explained by the results of chemical analysis, whereas for some of the more specific receptor-binding end points such as estrogenicity, a larger fraction of the effect can often be assigned to quantified chemicals [39, 40]. A recent interlaboratory comparison of bioassays using spiked extracts of pristine water unknown to the participants showed rather good agreement of results [37]. This demonstrates that like nontarget screening mentioned earlier, effect-based analysis is out of its infancy and is applicable in routine monitoring. Most bioassays focus on the measurement of short-term effects. Longer exposure in realistic exposure scenarios to cover long-term effects on organisms can be achieved by an in vivo test battery using flow-through systems [41].

If one wants to use the results of the bioassays to further direct the identification of causative chemicals, the sample or an extract can be fractionated, mostly by LC, and the individual fractions are investigated again. This approach is very time-consuming, and therefore an interesting alternative is a microfractionation system that combines LC or even LC×LC separations with a simultaneous collection of fractions in microtiter plates for effect analysis that allows for linking of effect and chemical identity [42–44]. The applicability of such a system has already been demonstrated for high-throughput screening of several end points relevant in aquatic systems. In all approaches, an important validation test is toxicity recovery (i.e., the test of whether the toxicity in combined fractions brought to the same volume as the initial sample equals the initial toxicity). If necessary, fractionation can be repeated, with the ultimate goal of isolating one or a few chemicals causing the toxic effect. In multistep fractionation it is advisable to make use of the different selectivities offered by different columns or even separation principles [36]. Fractionation procedures are not yet standardized, and therefore a diverse set of columns and elution conditions is used, which hampers comparison of data. Collaborative efforts in that regard would be important for the future. Complementary to the approach described is the use of thin-layer chromatography for a spatial separation of compounds. The thin-layer chromatography plates can then be directly used in biotest media to measure several end points, including estrogenicity [45] and acetylcholinesterase inhibition [46]. If an effect is found, it is visualized on the plate, and a further investigation of causative agents by off-line or online coupling with (HR)MS is much facilitated.

In summary, the field of bioanalytical tools complementary to advanced chemical analysis has matured over the past decade and should be equally considered in future regulation of required monitoring since both are needed to answer the pressing questions on the status of our aquatic systems. More and more we will also see a merger of both domains by combinations of high-throughput effect screening and HRMS use.

## Where else the area has to move on

As mentioned previously, the limitation of the scope of this article to OMP analysis is not meant to indicate that this is the only or even the most important area of research needs in water analysis. Thus, to finish, further topics of relevance are summarized in the following personal shortlist, again without the claim to be comprehensive:

- Culture-independent rapid identification and quantification of microorganisms including viruses
- Analysis of antibiotic resistance/antibiotic resistance genes and gene transfer in the environment
- Analysis of anthropogenic particles for abundance, type, and size in trace concentrations and with a high background of natural solids (nanomaterials, microplastics)
- Sensor networks for continuous monitoring and integrative sampling for the surveying of short-term (pulse) concentration changes
- Further refinement of still emerging analytical tools in water analysis such as compound-specific stable isotope analysis
- Element species analysis at trace concentrations in the environment: organically bound versus ionic (e.g., Hg, As, Sn), complexed (e.g., Gd), or redox sensitive (e.g., Cr(III) vs Cr(VI))
- Further automation of full analytical methods, including sample preparation
- Cost-effective in situ or on-site analysis, for example, by sensors or colorimetric tests using micro paper-based analytical devices and/or smartphones

## Outlook

As discussed in detail in the previous sections, the past few years have seen substantial developments in analytical science that have also reached the area of water analysis but are not yet established in routine monitoring or surveillance schemes. Foundations have been laid but this will be an important transfer task for the future. Although many scientists may not be very interested in related work, including the tedious task of standardization, it is of the utmost importance for long-lasting impact, including meaningful decisions for the future allocation of resources in water monitoring. In particular bringing together HRMS-based suspect and nontarget screening, on the one hand, and effect-based analysis, on the other hand, will be an important step forward in the field for its ultimate goal: to provide data that allow better protection of our invaluable water resources.
